# Conventional Complete Denture in Patients with Ectodermal Dysplasia

**DOI:** 10.1155/2015/714963

**Published:** 2015-09-06

**Authors:** Larissa Soares Reis Vilanova, Alfonso Sánchez-Ayala, Giselle Rodrigues Ribeiro, Camila Heitor Campos, Arcelino Farias-Neto

**Affiliations:** ^1^Department of Oral Health, Federal University of Goiás, Primeira Avenida, s/n, Goiânia, GO, Brazil; ^2^Department of Dentistry, State University of Ponta Grossa, Avenida General Carlos Cavalcanti, No. 4748, 84030-900 Ponta Grossa, PR, Brazil; ^3^Department of Prosthodontics and Periodontology, Piracicaba Dental School, University of Campinas, Avenida Limeira, No. 901, Piracicaba, SP, Brazil; ^4^Department of Dentistry, Health School, Potiguar University, Laureate International Universities, Avenida Senador Salgado Filho, No. 1610, Natal, RN, Brazil

## Abstract

Ectodermal dysplasia is described as heritable conditions that involve anomalies of structures derived from the ectoderm, including hypodontia. In the cases of edentulous young patients, who did not finish their craniofacial growth, treatment with conventional complete denture is a suitable alternative. The aim of this study was to report a case of mandibular edentulism treated with conventional complete denture in a thirteen-year-old patient diagnosed with hidrotic ectodermal dysplasia. Typical features, such as frontal bossing, depressed nasal bridge, protuberant lips, scarce hair, and brittle nails, were visualized during the extraoral examination. The intraoral inspection and radiographic analysis revealed oligodontia, dental malformation, and prolonged retention of deciduous teeth at maxilla and total edentulism at mandible. A conventional complete denture was planned and constructed following the same steps of technique as recommended in adults. Although this option is not a definitive treatment, the patient and his parents were satisfied with his improvement in chewing and speech, as well as with the aesthetic benefits.

## 1. Introduction

Ectodermal dysplasia (ED) is a large and heterogeneous group of congenital disorders that are categorized by defects in the development of tissues originated from the embryonic ectoderm [1]. This syndrome is relatively rare (seven cases/10,000 born) and its 150 subtypes are generally classified as hidrotic (Clouston's syndrome) or hypohidrotic (Christ-Siemens-Touraine syndrome) forms [2, 3]. ED can be inherited as sex-linked, autosomal dominant or autosomal recessive [3]. Spontaneous mutations may also be responsible for the appearance of this disease [4]. The clinical signals mainly include frontal bossing with prominent supraorbital ridges, more pronounced chin, broader noses and depressed nasal bridge, protuberant lips, thin, fragile, and sparse hair on the scalp and eyebrows, misshapen, brittle, and porous nails, oral abnormalities, and heat intolerance in the hypohidrotic variation by the absence of sweat glands [1, 2]. Abnormal immune response, functional abnormalities of central nervous system, mental retardation, skeletal conditions, cleft lip/palate, endocrine defects, retinal conditions, deafness, and skin diseases may also be seen [3].

The oral abnormalities include hypodontia (anodontia and oligodontia) of the primary and permanent teeth in booth maxillaries (predominantly in the mandible), enamel alterations, xerostomia, and all the maxillofacial changes and development consequences arising from lack of teeth, such as disturbances in form and size of the maxillary bones and alveolar ridges, malocclusions, soft tissue defects, reduced vertical dimension, and a typical aged appearance [1–4]. Despite the specific knowledge on diagnosis and genetic classification of ED, there is not a medical treatment or a specific treatment for edentulism in young patients [1]. However, certain prosthodontics procedures can improve the patient's oral function and appearance, at least until its cranial-facial growth is completed and implant-supported or retained dentures can be installed [2, 4]. It should be considered that ED patients usually are shy and withdrawn, and a dental treatment can rescue the self-esteem, sociability, and enjoyment of life. Therefore, the aim of the study was to report a case of mandibular edentulism treated with conventional complete denture in a thirteen-year-old patient diagnosed with hidrotic ectodermal dysplasia.

## 2. Case Report

**Figure 1:**
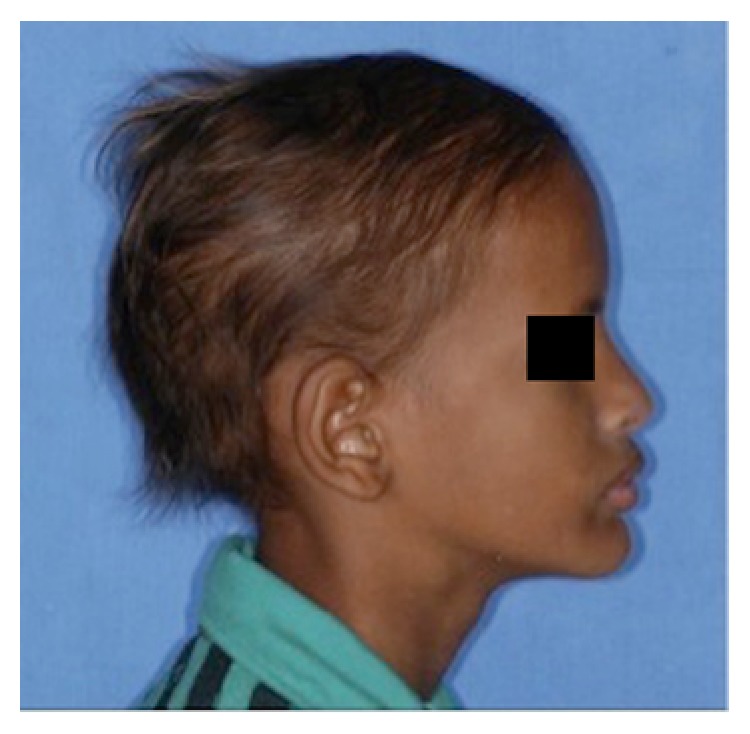
Facial profile view: sparse and fine hair, frontal bossing, high-set orbits, pronounced chin, prominent and reverse lips, sunken cheeks, and large and low set ears.

**Figure 2:**
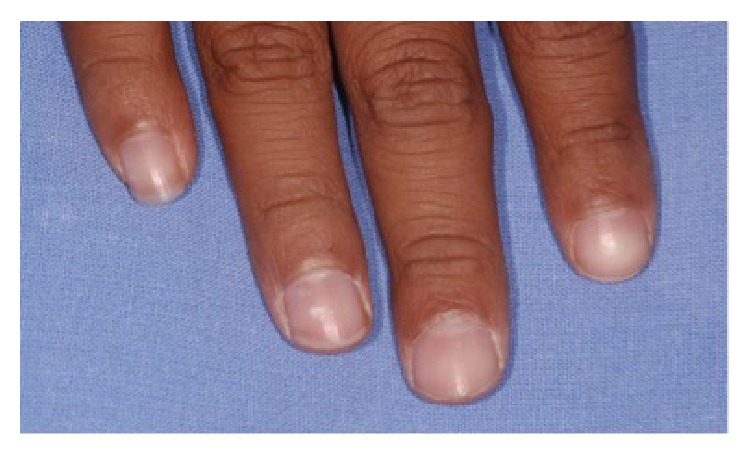
Brittle nails.

**Figure 3:**
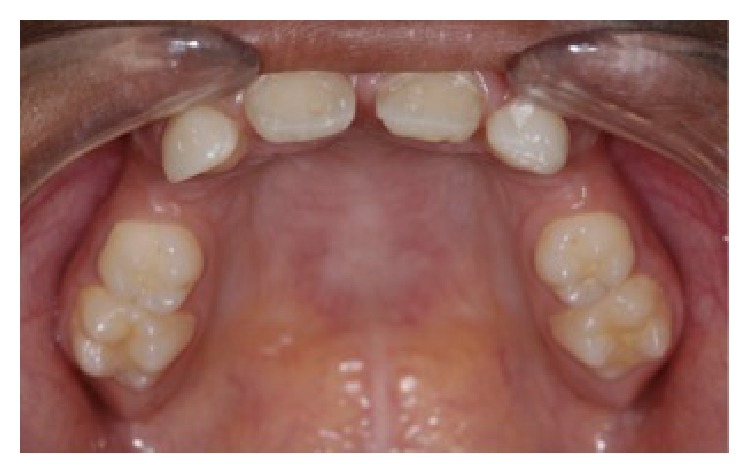
Intraoral view: mandibular edentulism and presence of permanent maxillary teeth 11, 16, 21, and 26 and deciduous teeth 52, 55, 62, and 65.

**Figure 4:**
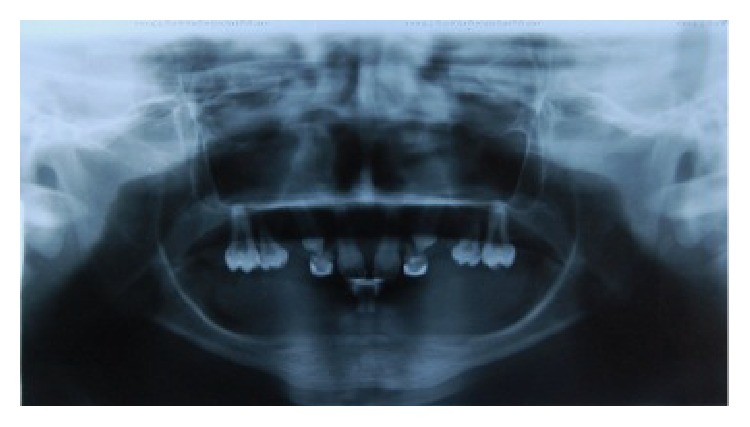
Panoramic radiograph view: prolonged retention of elements 52, 55, 62, and 65. The root resorption of teeth 52 and 62 can be observed, as well as the impaction of elements 13 and 23.

A 13-year-old male child diagnosed with hidrotic ectodermal dysplasia was referred to the Department of Dentistry at the Potiguar University. His parents related lack of teeth and difficulty in chewing as the chief complaint. According to report, despite the presence of some upper teeth, the lower ones never erupted. The body characteristics confirmed the diagnosis. The absence of mental, nervous, or maxillofacial disorders, as well as abnormal sweat, was also verified. On extraoral inspection, sparse and fine hair, frontal bossing, high-set orbits, pronounced chin, prominent and reverse lips, sunken cheeks, large and low set ears, and brittle nails were observed (Figures 1 and 2). Intraorally absence of all mandibular teeth, presence of teeth numbers 11, 16, 21, and 26 in the maxilla, and prolonged retention of deciduous teeth 52, 55, 62, and 65 were recognized (Figure 3). The panoramic radiograph confirmed the clinical data (Figure 4). Parents were sensitized about this condition, the maxillofacial and nutritional consequences, and the prosthodontics management according to age; therefore, a conventional complete denture was elected as treatment to mandible.

The technique to idealize a complete denture in children followed the same steps that are recommended for adults [2]. A preliminary impression was made using stock edentulous tray (Tecnodent, São Paulo, SP, Brazil) and irreversible hydrocolloid impression material Jeltrate (Dentsply, Milford, DE, USA). The final impression was taken with a custom tray fabricated with autopolymerized acrylic resin (Clássico, São Paulo, SP, Brazil) with border-moulded impression compound (Kerr, Orange, CA, USA), followed by a zinc oxide/eugenol impression Horus (Dentsply). Master casts performed by the use of type IV dental stone Durone (Dentsply) were mounted on a semiadjustable articulator with a common arbitrary ear-face-bow instrument, using condylar guidance of 30°, Bennett angle of 15°, and standard intercondylar distance (110 mm). Occlusal vertical dimension was established using the physiological rest positions associated with phonetic and esthetic techniques. The denture was made in centric occlusion with balanced articulation and anatomically shaped acrylic teeth with a cuspal inclination of 33° (Trubyte Biotone; Dentsply).

**Figure 5:**
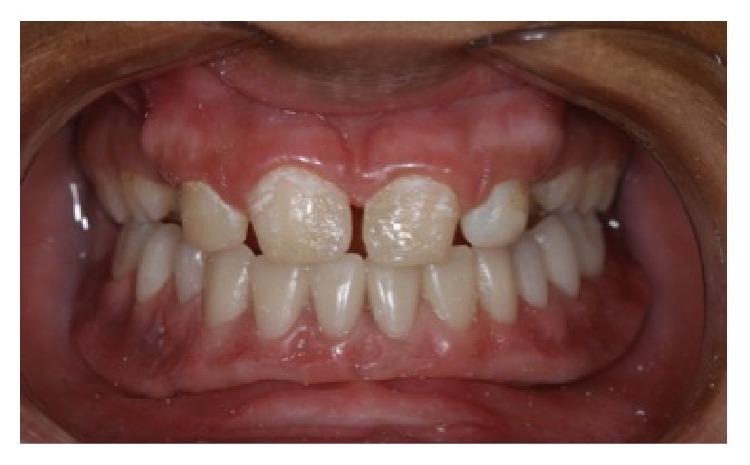
After prosthesis installation.

Centric occlusion was established according to dynamic records based on unforced movements of the jaws in the terminal hinge position performed by the patients and manually guided. As a previous clinical appraisal, the artificial teeth were arranged in wax. The patient and his parents accepted the arrangement of teeth. The dentures were waxed, processed, finished, and polished. After denture installation (Figure 5), the child and parents were counseled about its use, cleaning procedures, and the importance of followup, especially if there were some complaints of any discomfort. To complete necessary adjustments, a scheduling was done the next day and one and two weeks. The patient was monitored, verifying an improvement in physical and emotional state, with appropriate adaptation to new denture and subsequent progress in speech and esthetics. Future recall was also planned at 6 months to monitor bone growth and for eventual relining or construction of a new denture. A provisional maxillary removable partial denture and all-ceramic crowns or laminate veneers to central incisors will be proposed to parents after the complete eruption of canine teeth.

According to the panoramic radiograph (Figure 4), although the deciduous teeth 52 and 62 delayed their exfoliation (8-9 years) in the absence of dental germs corresponding to the permanent elements 12 and 22, apparently, the permanent canine teeth will stimulate their root resorption and subsequent exfoliation. However, since the normal age for eruption of teeth 13 and 23 can vary from 10 to 12 years, a slight delay in this process can also be considered. Therefore, orthodontic treatment was proposed in order to ensure the proper eruption of these elements, as well as plan their distal repositioning to create space for artificial lateral incisors. After that, a flexible removable partial denture to maxilla and fixed esthetic alternatives to teeth 11 and 21 can be performed. The remaining deciduous teeth 55 and 65 will be extracted due to their lower anatomical capacity for prosthodontic support and retention, as well as adult masticatory function. Considering the temporary nature of flexible denture, the biomechanical preparation of pillar teeth (13, 16, 23, and 26) should be minimal and prioritizing the adding of resin-based composite instead of enamel wear. Moreover, after completing the maxillary (around 15 years) and mandibular (around 20 or 21 years) growth, a removable or fixed implant therapy may be considered as final treatment.

## 3. Discussion

Treatment with implant-supported or retained dentures is the primary choice for teeth replacement and masticatory function rehabilitation. Cases reports have verified this alternative even in healthy young patients and those with ED [2, 5]. However, the use of implant therapy may fail due to the insufficient or lack of alveolar bone, embedding, relocation or displacement of implants by facial growth, eventual trauma to teeth germs, ankyloses teeth, and multidimensional restrictions of craniofacial growth [1]. Thus, to prevent these potential problems, the conventional complete denture employed in this report was an attempt to properly rehabilitate this patient and at least promote oral function and esthetic, prevent extrusion of antagonist teeth, and not impair the normal development of the mandibular and maxillary bones [2, 4]. In the case of oligodontia, partial dentures can also preserve the space created by the lack of deciduous teeth. Dentures in ED patients may be constructed even during preschool age, when children usually adapt easier to its use [2]. It is anticipated that as the patient continues to grow and mature, prosthetic replacements and periodic controls will be required.

Intraoral characteristics, such as reduced size of mandible arch and alveolar bone height with knife-edge shape, may complicate the denture construction and its biomechanical behavior during function [2]. An improved masticatory function can only be achieved by providing good denture stability and retention. An appropriate occlusal support also allows healthy temporomandibular joints and efficient mandibular kinematics. It should be considered that the absence of teeth and masticatory incapacity are fundamental factors to malnutrition, and the establishment of lifelong dietary patterns occurs during childhood [6]. Beside the satisfactory body development, an efficient masticatory function is related to postnatal brain development and learning. The muscular activity during mastication, regardless of calorie intake, increases the neuronal activity in the brain and cerebral blood flow [7]. Moreover, a correct vertical dimension of dentures can help not only prevent development mandibular of pseudoprognathism, but also improve skeletal relationship during growing period of the child [2, 4].

A trend of craniofacial growth towards a retrognathic maxilla and class III sagittal relationships of the jaws and a global reduction of all facial volumes in infancy before treatment were observed in ED patients [8]. After rehabilitation of ED adolescents, a larger growth than in controls was found in the width and height of the lower and upper facial third, while all facial depths and all distances in the middle facial third maintained an inferior growth [4]. At the end of adolescence, ED subjects showed similar growth patterns to controls of facial volumes and almost twice that of nonrehabilitated subjects [4, 8, 9]. The flat aged profile and frontal bossing may be consequence of diminished facial depths and retruded position of nasion, respectively [4]. It should be considered that the maxilla development depends on sutural growth, but the mandible mainly on endochondral growth at the condyles (which makes plausible the implant therapy in anterior mandible) [5]. However, although the growth of the mandibular symphysis may stop at 2 years, the fabrication of a new lower denture about every 6 months is suggested because transversal changes may still be detected [10].

## 4. Conclusion

The construction of a mandibular conventional complete denture allowed the maintenance of masticatory and phonetic functions, as well as an improvement in facial aesthetics. Patient satisfaction was evident through improved self-esteem and social reintegration.

## References

[1] Klineberg I., Cameron A., Whittle T. (2013). Rehabilitation of children with ectodermal dysplasia. Part 1: an international Delphi study. *The International Journal of Oral & Maxillofacial Implants*.

[2] Bidra A. S., Martin J. W., Feldman E. (2010). Complete denture prosthodontics in children with ectodermal dysplasia: review of principles and techniques. *Compendium of Continuing Education in Dentistry*.

[3] Priolo M., Laganà C. (2001). Ectodermal dysplasias: a new clinical-genetic classification. *Journal of Medical Genetics*.

[4] Dellavia C., Catti F., Sforza C., Tommasi D. G., Ferrario V. F. (2010). Craniofacial growth in ectodermal dysplasia. An 8 year longitudinal evaluation of Italian subjects. *Angle Orthodontist*.

[5] Filius M. A. P., Vissink A., Raghoebar G. M., Visser A. (2014). Implant-retained overdentures for young children with severe oligodontia: a series of four cases. *Journal of Oral and Maxillofacial Surgery*.

[6] Jain N., Naitam D., Wadkar A., Nemane A., Katoch S., Dewangan A. (2012). Prosthodontic rehabilitation of hereditary ectodermal dysplasia in an 11-year-old patient with flexible denture: a case report. *Case Reports in Dentistry*.

[7] Nakata M. (1998). Masticatory function and its effects on general health. *International Dental Journal*.

[8] Bondarets N., Jones R. M., McDonald F. (2002). Analysis of facial growth in subjects with syndromic ectodermal dysplasia: a longitudinal analysis. *Orthodontics & Craniofacial Research*.

[9] Dellavia C., Catti F., Sforza C., Grandi G., Ferrario V. F. (2008). Non-invasive longitudinal assessment of facial growth in children and adolescents with hypohidrotic ectodermal dysplasia. *European Journal of Oral Sciences*.

[10] Montanari M., Callea M., Battelli F., Piana G. (2012). Oral rehabilitation of children with ectodermal dysplasia. *BMJ Case Reports*.

